# Combination of transbronchial cryobiopsy based clinic-radiologic-pathologic strategy and metagenomic next-generation sequencing for differential diagnosis of rapidly progressive diffuse parenchymal lung diseases

**DOI:** 10.3389/fcimb.2023.1204024

**Published:** 2023-06-20

**Authors:** He Sun, Rongzhang Chen, Tian Li, Jinli Gao, Xia Gu, Xuyou Zhu, Lianfeng Jin, Yi Shi, Qiang Li

**Affiliations:** ^1^ Department of Respiratory and Critical Care Medicine, Shanghai East Hospital, Tongji University School of Medicine, Shanghai, China; ^2^ Department of Pathology, Shanghai East Hospital, Tongji University School of Medicine, Shanghai, China; ^3^ Department of Pathology, Tongji Hospital Affiliated to Tongji University, Shanghai, China; ^4^ Department of Scientific Affairs, Vision Medical Center for Infectious Diseases, Guangdong, China; ^5^ Department of Respiratory and Critical Care Medicine, Jinling Hospital, Clinical School of Nanjing, Nanjing, China

**Keywords:** rapidly progressive diffuse parenchymal lung diseases, transbronchial cryobiopsy, metagenomic next-generation sequencing, high-resolution computed tomography, pulmonary histopathology

## Abstract

**Background:**

The complicated spectrum of rapidly progressive diffused parenchymal lung diseases (RP-DPLD) creates obstacles to the precise diagnosis and treatment. We evaluated the differential diagnostic value of transbronchial cryobiopsy (TBCB) based clinic-radiologic-pathologic (CRP) strategy combined with bronchoalveolar lavage fluid (BALF) metagenomic next-generation sequencing (mNGS) in RP-DPLD patients.

**Methods:**

RP-DPLD patients who underwent the diagnostic strategy of TBCB-based CRP combined with BALF mNGS at Shanghai East Hospital from May 2020 to Oct 2022 were retrospectively analyzed. Clinical characteristics were summarized, including demographic data, high-resolution computed tomography (HRCT) findings, histopathology of TBCB and microbiological results. Diagnostic value of the combined strategy, as well as the sensitivity, specificity, and positive detection rates of mNGS were evaluated.

**Results:**

A total of 115 RP-DPLD patients were enrolled, with a mean age of 64.4 years old and a male proportion of 54.8%. The pulmonary imaging findings in most patients were complex and diverse, with all patients showing bilateral lung diffuse lesions in HRCT, and progressively aggravated imaging changes within one month. After combining TBCB-based CRP strategy with mNGS, all participants received a corresponding diagnosis with 100% diagnostic yield. In these patients, 58.3% (67/115) were diagnosed with noninfectious RP-DPLD and 41.7% (48/115) with infection-related RP-DPLD. There were 86.1% of cases with known etiology according to the DPLD classification. BALF mNGS and traditional pathogen detection methods were performed in all patients, the positive detection rates were 50.4% (58/115) and 32.2% (37/115), respectively. Meanwhile, the mNGS showed significantly higher sensitivity and negative predictive value than the traditional pathogen detection methods for the diagnosis of infection-related RP-DPLD (100% vs 60.4% (p<0.001), 100% vs 75.6% (p<0.001), respectively). Among noninfectious RP-DPLD patients, the true negative rate of mNGS was 85.1% (57/67). All patients had their treatment regimen modified and the 30-day mortality was 7.0%.

**Conclusion:**

The novel strategy of TBCB-based CRP combined with mNGS provided dependable and sufficient evidence for the diagnosis, meanwhile further improved the accuracy of RP-DPLD treatment, as well as the prognosis of patients. Our results highlight the significant value of combined strategy in determining whether the RP-DPLD patients were infection associated or not.

## Introduction

Diffuse parenchymal lung diseases (DPLD) are heterogeneous lung disorders presented with diffused lesions of pulmonary parenchyma on chest HRCT, which mainly involve pulmonary interstitium and alveolar cavity (Travis et al., 2013). Idiopathic interstitial pneumonia (IIP), connective tissue disease-associated interstitial (CTD-ILD), sarcoidosis and hypersensitivity pneumonitis (HP) are the common types of DPLD (Meyer, 2014). Rapidly progressive DPLD (RP-DPLD) exhibits deteriorating clinical presentations within one month, such as aggravated dyspnea, restrictive ventilatory dysfunction, reduced diffusion capacity and hypoxemia, which in turn lead to progressive loss of lung function, respiratory failure and eventually poor prognosis or even death within a short period of time (Mikolasch et al., 2017; Amundson et al., 2019). Therefore, accurate diagnosis of RP-DPLD is of significant importance for the prognosis of RP-DPLD.

Classical diagnostic modality of DPLD is based on clinic-radiologic-pathologic (CRP) pattern, including clinical evaluation, radiological imaging and pulmonary histopathology. The chest high-resolution computed tomography (HRCT) is the imaging reference standard and an essential part of the assessment of DPLD (Kifjak et al., 2022). Surgical lung biopsy (SLB), transbronchial forcep biopsy (TBFB), percutaneous lung biopsy (PNLB) and transbronchial cryobiopsy (TBCB) are main methods used to obtain histopathological specimens from specific parts of the lung parenchyma (Radu et al., 2012; Arya et al., 2020; Yoon et al, 2020; Ronaghi and Oh, 2022). SLB, due to the risks of thoracotomy, significant complication, great surgical trauma and increased costs, is not recommend in RP-DPLD patients (Molin et al., 1994; Hutchinson et al., 2016), even though it had been the gold standard of histopathology for the etiological diagnosis. TBFB and PNLB often provide insufficient samples, resulting in poor sensitivity and low diagnosis rate. However, acquisition of sufficiently large tissue with minimal injury is critical for precise diagnosis. Recent evidence demonstrated that the promising technique TBCB raised the diagnostic yield of DPLD (Lentz et al., 2018; Ravaglia et al., 2019). Therefore, TBCB-based CRP diagnostic strategy are gradually applied in clinical practice.

Even so, the available CRP evidence cannot provide adequate diagnostic information, resulting in a poor prognosis. A precise diagnosis remains challenging especially when confounders of infectious diseases are present. Previous research suggested that infection caused DPLD or co-infections following original DPLD are the common factors that trigger acute exacerbation of the disease (Vanfleteren and Linssen, 2010; Collard et al., 2016; Ye et al., 2020). Moreover, infections may play a considerable role in the complex interaction between a susceptible host and the environment, leading to the development or progression of interstitial lung disease (ILD) (Azadeh et al., 2017). Therefore, accurate and comprehensive pathogen information is of great significance for the precise treatment and better prognosis of RP-DPLD. Metagenomic next-generation sequencing (mNGS) has emerged as a comprehensive, high throughput tool for clinical infectious agent detection and noninfectious disorders identification (Wei et al., 2019; Zhou et al., 2022). However, there has been little discussion about the application of mNGS in RP-DPLD to date. Hence, we proposed TBCB-based CRP combined with mNGS as a novel strategy for differential diagnosis of RP-DPLD, providing more comprehensive and reasonable information about histopathology and pathogens. In this study, the application of TBCB-based CRP strategy combined with mNGS was evaluated in RP-DPLD patients.

## Methods

### Study subjects

This is a single center retrospective study involved 115 RP-DPLD patients admitted to the Department of Respiratory and Critical Care Medicine of Shanghai East Hospital from May 2020 to October 2022. The inclusion criteria for patients’ enrollment are as follows: 1) only adults (age≥18 years old) were included; 2) all subjects underwent thoracic HRCT and met the imaging features of DPLD, meanwhile all patients presented with radiographic aggravation in one month; 3) all patients were initially diagnosed as severe pneumonia and given anti-infective treatments before admitted to our department, but the disease continued to progress; 4) left heart failure or fluid overload could not be used for interpretation of the exacerbation of the disease; 5) all patients underwent TBCB, bronchoscopy biopsy brushes, and bronchoalveolar lavage; 6) all specimens were sent for pathological examination, traditional microbiological detection and mNGS. The present study excluded any subjects who could not tolerate the bronchoscopy procedure or patients with incomplete information.

### Specimens collection

Bronchoscopy (BF-260, Olmpus, Japan) was performed to collect specimens. Lung tissue specimens were obtained through TBCB under general anesthesia. Transbronchial brushing (TBBr) biopsy was collected via brushing the lung mucosa three times. Four flasks of bronchoalveolar lavage fluid (BALF) were collected using PBS buffer. After collection, one of the lung biopsy tissue was immediately fixed in formalin and sent for histopathology. TBBr biopsy was used for rapid on-site evaluation (ROSE). BALF samples were subjected to cytometric analysis and mNGS testing.

### Transbronchial cryobiopsy

TBCB method was carried out in accordance with Expert Consensus on Transbronchial Cryobiopsies (Medicine Branch of Interventional Respiratory et al., 2019). All patients underwent preoperative examinations such as blood routine, coagulation function, electrocardiogram, pulmonary function and chest HRCT. To avoid bleeding, antiplatelet agents were discontinued for 1 week before TBCB. TBCB was conducted in general anesthesia and the vital signs of patients were monitored.

### Metagenomic next-generation sequencing and bioinformatics analysis

BALF was collected and transported to perform mNGS detection. The mNGS and bioinformatics analysis were conducted similarly to our previous publication (Sun et al., 2022). The DNA was extracted and purified by taking 200 μL of BALF sample according to the instructions of QIAamp DNA Micro Kit (QIAGEN, Hilden, Germany). DNA concentration and quality were checked through Qubit 3.0 Fluoremeter (Invitrogen, Q33216) and agarose gel electrophoresis (Major Science, UVC1-1100). DNA library construction was performed according to the Qiagen library construction kit (QIAseq Ultralow Input Library Kit) operating instructions. Library quality control was performed by Qubit 3.0 Fluoremeter (Invitrogen, Q33216) and Agilent 2100 Bioanalyzer (Agilent Technologies, Palo Alto, USA). Qualified DNA libraries with different barcode tags were pooled and then sequenced using the Nextseq 550 platform (Illumina, San Diego, USA) and a SE 50bp sequencing strategy.

After obtaining the sequencing data, high quality data were generated by filtering out connectors, low quality, low complexity and shorter sequences. Next human-derived sequences matching to the human reference database (hg38) were removed by using SNAP software. The remaining data were then aligned to the microbial genome database using Burrow-Wheeler Alignment. The microbial composition of the samples was finally determined.

### Clinical data collection

Data collected included demographic data (gender, age, hospitalization time, ICU stay time etc.), underlying diseases, vital signs (oxygenation index, PaO_2_/FiO_2_), lab examination (white blood cell, WBC) count, platelet, C-reactive protein, procalcitonin (PCT), cytokine levels, imaging findings, histopathology features, microbiological identification (smear microscopy, culture, 1,3-β-D-Glucan testing, Gene Xpert of BALF, mNGS results), other clinical characteristics (non-invasive ventilation, NIV) and high-flow humidified oxygen, intubation and invasive ventilation (IV), ECMO, continuous renal replacement therapy (CRRT)), treatment, and prognosis.

### Diagnostic procedure of RP-DPLD

TBCB based CRP diagnostic workflow includes the following steps. Firstly, an exhaustive clinical history (past medical history, contact history, medication history, and smoking history) should be obtained, followed by laboratory investigations and pulmonary function tests. Secondly, chest HRCT are performed to collect the imaging features, which further help to define the etiology. Thirdly, a sufficiently large tissue is obtained through TBCB for histopathology, immunohistochemistry staining, and special stains in clinical practice. The clinical diagnosis of DPLD is determined based on multidisciplinary team (MDT) evaluation that includes clinical data, radiographic outcomes, and histopathology findings. In this study, all patients were diagnosed by combining TBCB-based CRP strategy with mNGS findings (Adegunsoye and Ryerson, 2021). Diagnoses were carried out based on relevant international or domestic diagnostic guidelines for these disease classification, including connective tissue disease-related interstitial lung disease (CTD-ILD) (Sjögren syndrome (SS), antisynthetase syndrome (ASS), anti-neutrophil cytoplasmic antibody associated vasculitis (AAV), eosinophilic pneumonia (EP), IgG4 related diseases (IgG4-RD), sarcoidosis, lymphangioleiomyomatosis (LAM), interstitial pneumonia with autoimmune features (IPAF), systemic lupus erythematosus (SLE), HP, drug-induced lung injuries (DLI), dermatomyositis (DM), eosinophilic pulmonary diseases (EPD), organizing pneumonia (OP), and idiopathic pulmonary fibrosis (IPF). Treatment was adjusted after definite diagnosis.

### Statistical analysis

SPSS 22.0 statistical software was applied for data processing, the counting data were expressed by case number and rate (%), and measurement data were expressed as mean ± standard deviation (SD). The Chi-squared (χ^2^) test was used for comparisons between groups, p < 0.05 was taken as statistically significant.

## Results

### Basic characteristics of the RP-DPLD patients

Between May 2020 and Oct 2022, 118 RP-DPLD patients were screened, and a total of 115 patients participated in the study. Of the patients enrolled, 63 (54.8%) were males and 52 (45.2%) were females, with a mean age of 64.41 ± 13.71 years (range: 18-92 years). All patients had various degrees of fever during the previous month, and presented aggravated dyspnea with cough. All enrolled patients had a history of anti-infective use within 30 days prior to enrollment. As for laboratory findings, the mean C-reactive protein, IL-6 and PCT increased in all patients. All showed a decreased oxygenation index, including 38 (33.04%) cases with an oxygenation index between 200 and 300 mmHg; 59 (51.30%) between 100 and 200 mmHg; 18 (15.66%) below 100 mmHg. Ninety (78.26%) patients had been on high-flow humidified oxygen and non-invasive ventilation, and 21 (18.26%) patients received intubation and invasive ventilation. CRRT was used in four (3.48%) patients, and ECMO was used in three (2.61%). The baseline values are given in **Table 1**.

**Table 1:** Baseline characteristics of RP-DPLD patients.

Characteristics	All patients (n=115)	Infection-related RP-DPLD (n=48)	Noninfectious RP-DPLD (n=67)	*p*-value
Sex, male, n (%)	63 (54.8%)	28 (58.33%)	35 (52.23%)	>0.05
Age, mean ( ± SD)	64.41 ± 13.71	67.73 ± 15.30	62.03 ± 12.00	>0.05
C-reactive protein (mg/L), mean ( ± SD)	66.64 ± 67.94	91.3 ± 72.48	47.73 ± 58.05	<0.001
IL-6 (pg/mL), mean ( ± SD)	156.67 ± 333.8	260.05 ± 438.81	68.9 ± 166.77	<0.05
PCT (ng/mL), mean ( ± SD)	3.21 ± 11.94	5.86 ± 16.69	0.86 ± 3.74	<0.05
200<P/F ≤ 300 mmHg, n (%)	38 (33.04%)	15 (31.25%)	23 (34.32%)	>0.05
100<P/F ≤ 200 mmHg, n (%)	59 (51.30%)	25 (52.08%)	34 (50.74%)	>0.05
P/F ≤ 100 mmHg, n (%)	18 (15.66%)	8 (16.67%)	10 (14.92%)	>0.05
NIV and high-flow humidified oxygen, n (%)	90 (78.26%)	35 (72.91%)	55 (82.08%)	>0.05
Intubation and IV, n (%)	21 (18.26%)	9 (27.08%)	12 (17.91%)	>0.05
Vasoconstrictor, n (%)	33 (28.70%)	18 (37.5%)	15 (22.38%)	>0.05
ECMO, n (%)	3 (2.61%)	1 (2.08%)	2 (2.98%)	>0.05
CRRT, n (%)	4 (3.48%)	3 (6.25%)	1 (1.49%)	>0.05

Data are presented as average ± SD or numbers (percentages). RP-DPLD, rapidly progressive diffuse parenchymal lung diseases; PCT, procalcitonin; P/F, oxygenation index (PaO_2_/FiO_2_); NIV, non-invasive ventilation; IV, invasive ventilation; CRRT, continuous renal replacement therapy.

Although all patients had bilateral lung diffuse lesions confirmed by chest HRCT, the imaging findings presented distinct diversity and complexity. Most chest HRCT revealed increased lung markings, coarsening and disorder in both lungs, with some showing a grid image, stripe and nodular shadow, or cystoid shadow. Majority of the RP-DPLD patients with underlying diseases and infection presented acute exudation and consolidation, with partly pleural effusion. The imaging characteristics of different RP-DPLD patients were summarized in **Supplementary Table 1.**

### Differential diagnosis of RP-DPLD

**Figure 1:**
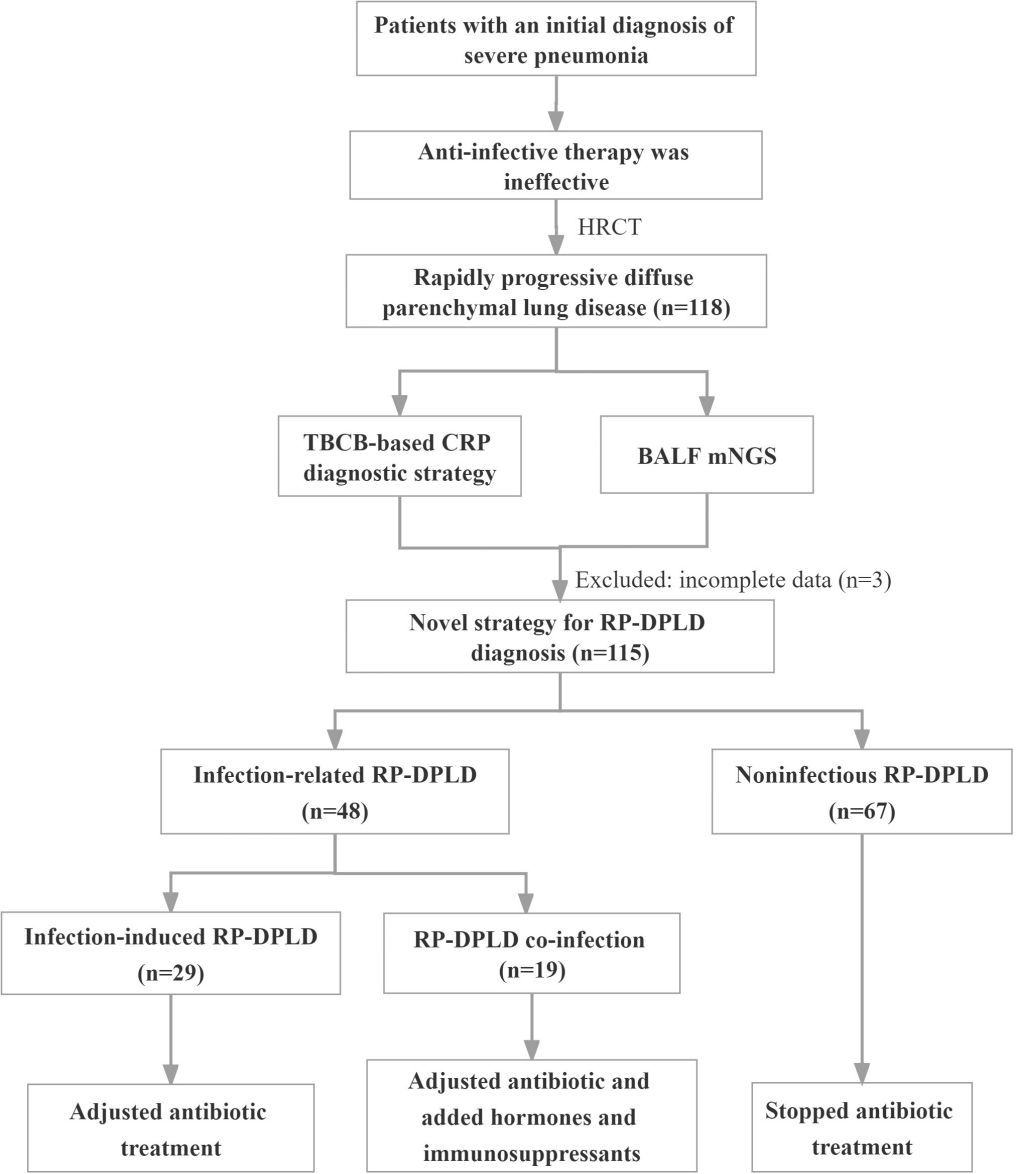
The diagnostic flow chart.

**Figure 2:**
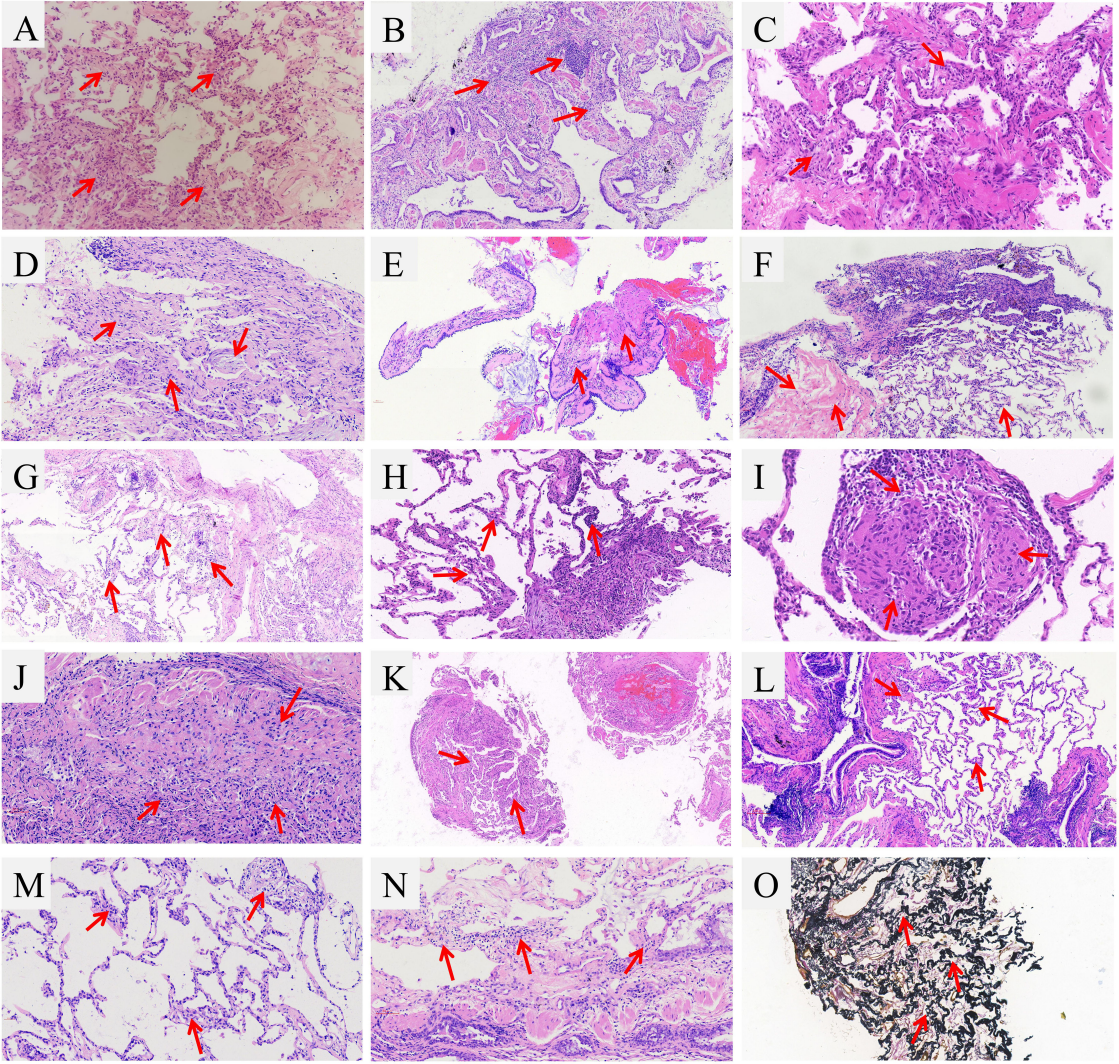
Representative images of TBCB of RP-DPLD patients. **(A)** SLE with SS and co-infection patient showed a chronic inflammation of the lung tissue, focal lymphocytic infiltration, widening of alveolar septum and capillary distention with hyperplasia of elastic fibers, macrophage accumulation in part of alveolar cavity; **(B)** Patient of dermatomyositis and co-infection showed a chronic inflammation and fibrosis, localized fibroblastic proliferation, protruding into the luminal surface of coated respiratory epithelium; **(C)** Patient with AAS exhibited the pulmonary septum was widened, mild hyperplasia of alveolar epithelial cells, as well as cellulose-like exudate, lymphocytes and foamy histiocytes were visible in alveolar cavities; **(D)** Biopsy of SS patient presented focal organizing pneumonia, atypical hyperplasia of alveolar epithelium and fibrous thickening of interalveolar septa; **(E)** EPD patient showed a chronic bronchiolitis with peritubula fibrous tissue hyperplasia and collagenization, focal infiltrates of eosinophils; **(F)** LAM patient showed a multifocal hyperplasia of interstitial immature smooth muscle cells, and cystic lumen was seen; **(G)** AAV patient showed a vascular malformation and proliferation, organizing pneumonia was seen in peripheral lesions, macrophage accumulation in the alveolar space; **(H)** Patient with IgG4-RD showed a fibrous tissue hyperplasia in the focal alveolar septum along with infiltration by lymphocytes and plasma cells, the ratio of IgG4-positive cells to plasma cells was 30%; **(I)** Sarcoidosis patient exhibited a few granuloma tissue formed in the lung, but necrosis was not found; **(J)** Patient of amiodarone-induced lung injury exhibited a fibrous hyperplasia in the bronchiolar walls along with inflammatory cell infiltration, peripheral lung and interstitial fibrosis, as well as focal vascular sclerosis; **(K)** Patient with PD-1-related pneumonitis revealed a few lung tissue and alveolar septum were slightly widened and a small amount of lymphocyte infiltration was observed; **(L)**. HP patient showed the bronchiolar lumen occlusion and peribronchial lymphocyte aggregates; **(M)**. Patient with pulmonary lymphoma showed a interstitial round cells in vessels wall and interstitium; **(N)**. Patient with smoking related ILD showed a localized organizing pneumonia around the pneumatic cavity; **(O)**. IPPFE patient showed a chronic inflammation in the lungs with tissue hyperplasia of elastic fibers (elastic fiber staining).

Combining TBCB-based CRP strategy with mNGS to establish a final diagnosis, all diagnoses were based on relevant international or domestic diagnostic guidelines. In this study, all participants received a corresponding diagnosis, the diagnostic yield was 100%. All patients were divided into two groups according to whether or not they have an infection, 67 (58.3%) patients were classified as the noninfectious RP-DPLD and 48 (41.7%) as the infection-related RP-DPLD. The diagnostic flow chart is shown in **Figure 1**. Further diagnostics were performed according to the etiologic classification of DPLD, and 16 (13.9%) cases were of unknown cause of RP-DPLD, including 12 patients with IPAF, 3 with OP and 1 with IPPFE. Among 86.1% of cases with known etiology, CTD-ILD (n=17, 14.8%) was the most noninfectious RP-DPLD followed by metastatic malignant tumors of the lung (n=13, 11.3%), HP (n=10, 8.7%), DLI (n=7, 6.1%) and others (n=4, 3.6%). The infection-related RP-DPLD (n=48, 41.7%) was further divided into two subgroups, infection-induced RP-DPLD (n=29, 25.2%) and RP-DPLD co-infection (n=19, 16.5%) (13 with CTD, 6 with IPF). These patients had distinct histopathological characteristics and clinical features; the representative histopathological findings are shown in **Figure 2**. **Table 2** shows the final diagnoses and histopathological features in RP-DPLD patients.

**Table 2:** Diagnoses and histopathological features of RP-DPLD patients.

Group	Disease category	Disease subtypes	Case No.	Histopathological description of representative patient
**Infection-related RP-DPLD**	Infection-induced RP-DPLD		29	/
	CTD-ILD co-infection	SLE with SS	2	Chronic inflammation of the lung tissue, focal lymphocytic infiltration, widening of alveolar septum and capillary distention with hyperplasia of elastic fibers, macrophage accumulation in part of alveolar cavity.
		Vasculitis	4	Chronic inflammation of the vascular wall with a large number of neutrophil infiltration.
		DM	3	Chronic inflammation and fibrosis, localized fibroblastic proliferation, protruding into the luminal surface of coated respiratory epithelium.
		Rheumatoid arthritis	4	The alveolar septum was not significantly widened and focal aggregation of few histocytes in the alveolar space, small foci of fibrosis, infiltration by a small amount of lymphocytes and plasma cells within the interstitium.
	IPF co-infection		6	Localized organizing pneumonia in the alveolar lumen and atypical granulomas were seen.
**Noninfectious RP-DPLD**	CTD-ILD	SS	2	Presented with focal organizing pneumonia, atypical hyperplasia of alveolar epithelium and fibrous thickening of interalveolar septa.
snf		AAS	7	The pulmonary septum was widened, mild hyperplasia of alveolar epithelial cells, as well as cellulose-like exudate, lymphocytes and foamy histocytes were visible in alveolar cavities.
		AAV	2	Vascular malformation and proliferation; organizing pneumonia was seen in peripheral lesions, macrophage accumulation in the alveolar space.
		EPD	2	Chronic bronchiolitis with peritubula fibrous tissue hyperplasia and collagenization, focal infiltrates of eosinophils.
		IgG4-RD	1	Fibrous tissue hyperplasia in the focal alveolar septum along with infiltration by lymphocytes and plasma cells, the ratio of IgG4-positive cells to plasma cells was 30%.
		Sarcoidosis	2	A few granuloma tissue formed in the lung, necrosis was not found.
		LAM	1	Multifocal hyperplasia of interstitial immature smooth muscle cells, and cystic lumen was seen.
	DLI	Amiodarone-induced lung injury	3	Fibrous hyperplasia in the bronchiolar walls along with inflammatory cell infiltration, peripheral lung and interstitial fibrosis, as well as focal vascular sclerosis.
		PD-1	3	A few lung tissue and alveolar septum were slightly widened and a small amount of lymphocyte infiltration was observed.
		Paraquat	1	Chronic inflammation of a few bronch and lung tissue.
	Metastatic malignant tumor of the lung		13	Visible tumor nodules.
	IPAF		12	Alveolitis, localized organizing pneumonia.
	HP		10	Bronchiolar lumen occlusion and peribronchial lymphocyte aggregates.
	OP		3	Vascular malformation with bleeding, cellulose exudation of alveolar cavities, with organizing pneumonia.
	Pulmonary lymphoma		2	Interstitial round cells in vessels wall and interstitium was observed.
	Smoking related ILD		1	Hyperplasia of the bronchiole mucosa epithelium, dilated lumens, the small blood vessels hyperplasia, localized organizing pneumonia around the pneumatic cavity.
	Radiation pneumonitis		1	A focal pseudostratified columnar ciliated epithelium was observed and hyperplasia of alveolar epithelium, chronic inflammation.
	IPPFE		1	Chronic inflammation in the lungs with hyperplasia of elastic fibers.

Data are presented as numbers. RP-DPLD, rapidly progressive diffuse parenchymal lung diseases; SLE, systemic lupus erythematosus; SS, Sjögren syndrome; DM, dermatomyositis; IPF, idiopathic pulmonary fibrosis; CTD-ILD, connective tissue disease-related interstitial lung disease; HP, hypersensitivity pneumonitis; ASS, antisynthetase syndrome; AAV, anti-neutrophil cytoplasmic antibody associated vasculitis; EPD eosinophilic pulmonary diseases; IgG4-RD, IgG4 related diseases; LAM, lymphangioleiomyomatosis; IPAF, interstitial pneumonia with autoimmune features; DLI, drug-induced lung injuries; OP, organizing pneumonia; ILD, interstitial lung disease; IPPFE, idiopathic pleuroparenchymal fibroelastosis.

"/" represents "not available".

The clinical characteristics of infection-related group and noninfectious group were evaluated. There were no significant difference between the two groups in terms of age, gender, oxygenation index, ventilation and vasoactive medication. However, the serum C-reactive protein, IL-6, PCT level of infection-related group were significantly higher than that of noninfectious RP-DPLD patients (all p<0.05) (**Table 1**).

### Diagnostic efficacy of mNGS

**Figure 3:**
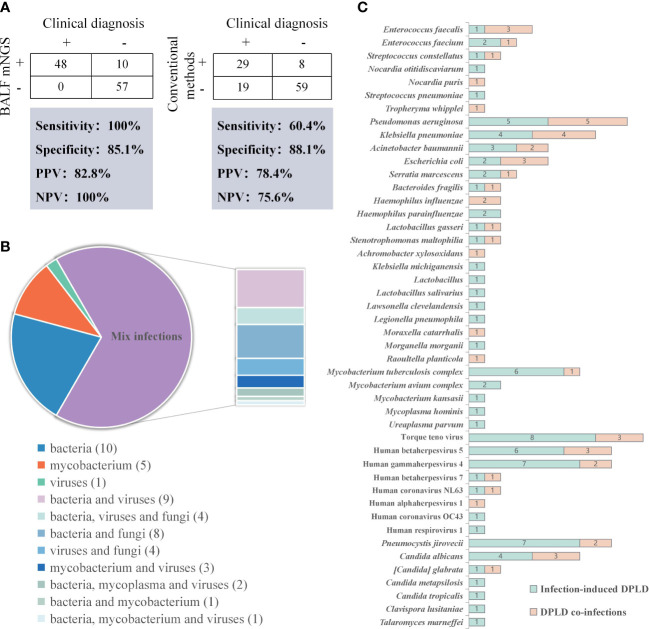
Results of the pathogenic microorganisms for RP-DPLD patients. **(A)**. Contingency tables for the clinical diagnosis with BALF mNGS and conventional methods sets, respectively; **(B)**. Classification of infection type by mNGS detection in infection-related RP-DPLD patients; **(C)**. The distribution of predominant pathogens in infection-related RP-DPLD patients.

BALF mNGS and traditional pathogen detection methods were performed in all RP-DPLD patients, and the positive detection rates were 50.4% (58/115) and 32.2% (37/115), respectively. The sensitivity, specificity, positive predictive value (PPV) and negative predictive value (NPV) of mNGS was 100%, 85.1%, 82.8% and 100%, respectively. The diagnostic efficacy of mNGS were compared to that of traditional pathogen detection methods. The mNGS showed significantly higher sensitivity and NPV than the traditional pathogen detection methods (100% vs 60.4%, 100% vs 75.6%, both p<0.001) (**Figure 3A**).

Among the 48 infection-related patients, all patients had positive mNGS results with 100% accuracy. Further analysis of 67 noninfectious RP-DPLD revealed that the true negative rate of mNGS was 85.1% (57/67), indicating that mNGS can efficiently distinguish noninfectious RP-DPLD from infection-related group. Notably, all negative results of mNGS were true negative patients and NPV was 100% (57/57). The leading diagnoses of true negative patients were CTD-ILD (n=13), metastatic malignant tumors of the lung (n=12), IFAF (n=12), HP (n=10), DLI (n=5), OP (n=2), smoking related ILD (n=1), pulmonary lymphoma (n=1), and IPPFE (n=1). These data suggest that mNGS have value for distinguishing noninfectious RP-DPLD.

Moreover, in the infection-related group, bacterial infection was identified in 10 (20.83%) patients, virus infection in 1 (2.08%) patient, mycobacterium infection in 5 (10.42%) patients, and mixed infections in 32 (66.67%) patients, including bacteria-virus (n=9), bacteria-fungi (n=8), bacteria-virus-fungi (n=4), virus-fungi (n=4), mycobacterium and viruses (n=3), and other mixed infections (n=4). The proportion of mixed infections was significantly higher than that of single bacteria, virus or mycobacterium infection (**Figure 3B**). *Pseudomonas aeruginosae* (n=10) was the most commonly detected bacterium, followed by *Klebsiella pneumonia* (n=8), *Mycobacterium tuberculosis complex* (n=7), *Acinetobacter baumannii* (n=5), and *Escherichia coli* (n=5). Torque teno virus (TTV) (n=11), Human gamma herpes virus 4 (EBV) (n=9) and Human beta herpes virus 5 (CMV) (n=9) were the most frequently detected viruses. The most common fungus was *Pneumocystis jirovecii* (n=9), followed by *Candida albicans* (n=7), and other fungi (n=6). We conducted pathogen spectrum comparisons between infection-induced RP-DPLD subgroup and RP-DPLD co-infection subgroup, and found that infection-induced RP-DPLD subgroup had a higher frequency of *Mycobacterium tuberculosis complex*, *Pneumocystis jirovecii*, TTV, EBV, CMV (**Figure 3C**).

### Treatment strategies and prognosis

After differential diagnosis by TBCB-based CRP strategy combined with mNGS, all patients had their treatment regimen modified. Sixty-seven (58.3%) cases stopped empiric antibiotic therapy, twenty-nine (25.2%) adjusted antibiotic regimen, nineteen (16.5%) adjusted antibiotics plus hormones and immunosuppressant therapies. The relevant information of treatment regimens is shown in **Supplementary Table 2**. Average total hospital stay was 15.6 ± 11.7 days, and stay in the ICU for patients requiring aggressive monitoring (n=41, 35.7%) averaged 14.3 ± 10.7 days. ICU admission for patients with infection-related RP-DPLD had a higher proportion than the noninfectious group (68.8% vs 11.9%; P<0.05). Four patients (3.5%) had been cured, and the conditions of 103 (89.5%) improved. Eight (7.0%) patients died within 30 days of confirmed diagnosis. Regarding the clinical outcomes, the noninfectious RP-DPLD exhibited a significant higher number of improved patients compared with the infection-related group (97.0% vs 79.2%; P<0.05). The medication adjustments and clinical outcomes are provided in **Table 3**.

**Table 3:** The medication adjustments and clinical outcomes of RP-DPLD patients.

Clinical treatments and outcomes	All patients (n=115)	Infection-related RP-DPLD (n=48)	Noninfectious RP-DPLD (n=67)	*p*-value
Stopped empiric antibiotic therapy, n (%)	67 (58.3%)	**/**	/	/
Adjusted antibiotic regimen, n (%)	29 (25.2%)	**/**	/	/
Adjusted antibiotics plus hormones and immunosuppressants, n (%)	19 (16.5%)	/	/	/
Hospitalization days, mean ( ± SD)	15.6 ± 11.7	17.5 ± 14.0	14.2 ± 9.6	>0.05
Admitted to ICU, n (%)	41 (35.7%)	33 (68.8%)	8 (11.9%)	<0.05
Cured, n (%)	4 (3.5%)	4 (8.3%)	0	>0.05
Improved, n (%)	103 (89.5%)	38 (79.2%)	65 (97.0%)	<0.05
30-day case fatality, n (%)	8 (11.3%)	6 (12.5%)	2 (3.0%)	>0.05

Data are presented as average ± SD or numbers (percentages).

"/" represents "not available".

## Discussion

This study demonstrated the utility of TBCB-based CRP strategy combined with mNGS for the diagnosis of RP-DPLD and for the antibiotic stewardship. It suggested that TBCB can assist clinicians to obtain high-quality lung specimens for histopathological examination. In addition, concomitant usage of TBCB-based CRP strategy and mNGS may distinguish infection from non-infection and identify the causative pathogen to reduce the misuse of antibiotics.

RP-DPLD is a life-threatening event with poor prognosis and high mortality, as high as 43.9% (Sato et al., 2014). Acute exacerbation of IPF patients had high mortality rate of 50%~80%, and the median survival was approximately 3~4 months (Collard et al., 2016). Except for this, patients with RP-DPLD can develop acute alveolar damage, pulmonary edema and inflammation and progress into acute respiratory distress syndrome (ARDS), which leads to a high mortality rate (Rubenfeld et al., 2005). Early diagnosis and timely treatment are keys to reducing the mortality associated with DPLD. As a consequence, we sought to find an efficient approach for the diagnosis of RP-DPLD. TBCB-based CRP strategy is widely used in the diagnosis of DPLD, the use of which has been standardized through expert recommendations (Hetzel et al., 2018). Prior studies that have evaluated the validity of TBCB for diagnosing DPLD, with the overall diagnostic yield of 80% (Kropski et al., 2013; Bondue et al., 2017). However, most of the studies were conducted on noninfectious DPLD diagnosis, there was little literature on the application of TBCB-based CRP strategy in the diagnosis of all DPLD including infection-related and non-infection related DPLD. To our knowledge, this study was the first to describe the approach of TBCB-based CRP combined with mNGS for differential diagnosis in patients with infection-related or noninfectious RP-DPLD. The novel strategy on RP-DPLD patients showed a diagnostic yield of 100%, higher than previously reported. Notably, the mortality rate in this study was 7%, which is much lower than those in some results reported before. Together, the new combined strategy demonstrated a solid diagnostic performance.

Some of the patients achieved a definitive diagnosis through a particular histopathological feature. However, in a considerable proportion of the cases, no infectious agents or other etiologies can be determined and the DPLD is then classified as idiopathic. Recently, mNGS played a major role in detecting pathogens and confirming the etiology, especially combining with ROSE, endobronchial ultrasound-guided transbronchial lung biopsy and other methods (Li et al., 2020; Zhang et al., 2021). Infection-related RP-DPLD (41.7%) was the most diagnosis by performing the novel strategy in our study. BALF mNGS demonstrated a high sensitivity (100%) and PPV (82.8%) in diagnosing infection-related RP-DPLD patients, with the sensitivity higher than previous reports of 61.11%~97.1% (Liu et al., 2019; Hongbin et al., 2020; Jiang et al., 2022). Meanwhile, the results demonstrate that the sensitivity of mNGS is better than conventional detection techniques, we believed that mNGS is appropriate for diagnosis of RP-DPLD.

The abuse of antibiotics is a universal problem. Over half of the antibiotic prescriptions were inappropriate in secondary-level and tertiary-level hospitals in China, suggesting urgent measures are necessary to restrict the events (Zhao et al., 2021). Exclusion of infection remains important. Ma et al. explored the clinical significance of negative results of BALF mNGS, demonstrated that negative results of mNGS can help to exclude infection (Ma et al., 2022). *Qian et al.* evaluated the clinical value of negative mNGS results and found that when the mNGS test is negative, the negative prediction accuracy rate of the original specimen is significant (Qian et al., 2022). In our study, all patients were initially diagnosed with pneumonia and given anti-infective treatments before admitted to our department. The final diagnosis of all patients was revised after using the new diagnostic modality. Therein, mNGS exhibited a high specificity (85.1%) and NPV (100%) in diagnosing noninfectious RP-DPLD patients, and 58.3% of cases stopped empiric antibiotic therapy. Our data suggested that mNGS had a good diagnostic value in excluding infections of RP-DPLD, and rapid diagnostics of noninfectious illness by mNGS reduced empiric antibiotic use.

Viral, bacterial, or mycoplasma infections are suggested to be one of the triggers for DPLD. A prospectively study found that viral infections (CMV and HHV-7) were identified in both patients with acute exacerbation of IPF and non-IPF ILDs, but the clinical significance on short-term mortality remains undetermined (Saraya et al., 2018). In a retrospective review by *Xu*, multiple pathogen infection occupied approximately 60% in patients with infectious interstitial pneumonia, and the most common pathogens were G- bacteria, followed by *mycoplasma pneumoniae*, G+ bacteria, fungus and CMV (Xu, 2020). Several reports have shown that, in some situation, patients with an acute exacerbation of interstitial lung disease are caused by a viral infection, such as VZV or Enterovirus D68 (Vassallo, 2003; Matsumoto et al., 2016; Ueno et al., 2021). Our findings enriched the pathogen spectrum in infection-related RP-DPLD. The percentage of mixed infections reaches up to 66.7%, G- bacteria and virus were predominant among infections, which was consistent with previous reports. Surprisingly, *Mycobacterium* and *Pneumocystis jirovecii* were detected multiple times and were recognized as an important pathogens, especially in infection-induced RP-DPLD. Also, *Aspergillus* was not observed in our study, we guessed that the cause may be due to small number of patients enrolled and population selection. Further study is needed to explore the pathogenic characteristics.

There was an increased risk of ICU admission in patients with infection-related RP-DPLD. This finding was similar to that reported by *Song et al.*, in which they suggested that infection is a frequent factor for IPF patient with rapid deterioration (Song et al., 2011). Moreover, the prognosis of infection-related RP-DPLD were worse than those of noninfectious RP-DPLD, indicating a significant impact of infection on the outcome in RP-DPLD patients. This means that clinicians should pay more attention to the detection of infectious agents in RP-DPLD patients. We are convinced that the novel diagnostic methods we presented from this study could be successfully used in differential diagnosis of RP-DPLD, improving the prognosis of these patients.

## Limitations

The present study must be cautiously interpreted with respect to some limitations. Firstly, there is no uniform standard for interpretation of mNGS results, and the judgment of causative pathogen was carried out in accordance with clinical experience. Secondly, the present study did not include a separate detection control group, there were no comparative cohorts between combined diagnosis and separate methods. Lastly, this was a single-center retrospective study and there might be selection bias, and prospective, multi-centered, large-scale studies are warranted.

## Conclusions

Our study indicated that TBCB-based CRP strategy combined with mNGS presented excellent feasibility for RP-DPLD of rapidly and accurately diagnosis. We highlight the importance of the novel strategy for diagnosing infection-related and noninfectious RP-DPLD. Meanwhile, the novel diagnostic strategy had an instructive effect on the optimization of treatment regimen, and it appears to have significantly decreased mortality of RP-DPLD.

## Data availability statement

The datasets presented in this study can be found in online repositories. The names of the repository/repositories and accession number(s) can be found in the article/**Supplementary Material**.

## Ethics statement

Written informed consent was obtained from the individual(s) for the publication of any potentially identifiable images or data included in this article.

## Author contributions

HS, YS and QL designed the paper and guided the implementation. HS drafted the manuscript. HS, RC, TL, XG, JG, and XZ, collected the cases and summarized the data. LJ processed the data and did the statistical analysis. All authors approved the final manuscript as submitted and agree to be accountable for all aspects of the work.

## Glossary

**Table:**

DPLD	diffuse parenchymal lung diseases
RP-DPLD	rapidly progressive diffuse parenchymal lung diseases
IIP	idiopathic interstitial pneumonia
CTD-ILD	connective tissue disease-related interstitial lung disease
HP	hypersensitivity pneumonitis
CRP	clinic-radiologic-pathologic
HRCT	high-resolution computed tomography
SLB	surgical lung biopsy
TBFB	transbronchial forcep biopsy
PNLB	percutaneous lung biopsy
TBCB	transbronchial cryobiopsy
ILD	interstitial lung disease
mNGS	metagenomic next-generation sequencing
TBBr	transbronchial brushing
BALF	bronchoalveolar lavage fluid
ROSE	rapid on site evaluation
P/F	oxygenation index (PaO_2_/FiO_2_)
WBC	white blood cell
PCT	procalcitonin
NIV	non-invasive ventilation
IV	invasive ventilation
CRRT	continuous renal replacement therapy
MDT	multi-disciplinary team
SS	Sjögren syndrome
ASS	antisynthetase syndrome
AAV	anti-neutrophil cytoplasmic antibody associated vasculitis
EP	eosinophilic pneumonia
IgG4-RD	IgG4 related diseases
LAM	lymphangioleiomyomatosis
IPAF	interstitial pneumonia with autoimmune features
SLE	systemic lupus erythematosus
DLI	drug-induced lung injuries
DM	dermatomyositis
EPD	eosinophilic pulmonary diseases
OP	organizing pneumonia
IPPFE	idiopathic pleuroparenchymal fibroelastosis
IPF	idiopathic pulmonary fibrosis
SD	standard deviation
TTV	Torque teno virus
EBV	Human gamma herpes virus 4
CMV	Human beta herpes virus 5
ARDS	acute respiratory distress syndrome

## References

[B1] Adegunsoye A. Ryerson C. J. (2021). Diagnostic classification of interstitial lung disease in clinical practice. Clin. Chest Med. 42 (2), 251–261. doi: 10.1016/j.ccm.2021.03.002 34024401

[B2] Amundson W. H. Racila E. Allen T. Dincer H. E. Tomic R. Bhargava M.. (2019). Acute exacerbation of interstitial lung disease after procedures. Respir. Med. 150, 30–37. doi: 10.1016/j.rmed.2019.02.012 30961948

[B3] Arya R. Boujaoude Z. Rafferty W. J. Abouzgheib. Usefulness W. (2020). And safety of transbronchial biopsy with large forceps during flexible bronchoscopy. Proc. (Bayl Univ Med. Cent) 34 (2), 232–236. doi: 10.1080/08998280.2020.1835123 33678954 PMC7901402

[B4] Azadeh N. Limper A. H. Carmona E. M. Ryu J. H. (2017). The role of infection in interstitial lung diseases: a review. Chest 152 (4), 842–852. doi: 10.1016/j.chest.2017.03.033 28400116 PMC7094545

[B5] Bondue B. Pieters T. Alexander P. De Vuyst P. Ruiz Patino M. Hoton D.. (2017). Role of transbronchial lung cryobiopsies in diffuse parenchymal lung diseases: interest of a sequential approach. Pulm Med. 2017, 6794343. doi: 10.1155/2017/6794343 28512583 PMC5415669

[B6] Collard H. R. Ryerson C. J. Corte T. J. Jenkins G. Kondoh Y. Lederer D. J.. (2016). Acute exacerbation of idiopathic pulmonary fibrosis. an international working group report. Am. J. Respir. Crit. Care Med. 194 (3), 265–275. doi: 10.1164/rccm.201604-0801CI 27299520

[B7] Hetzel J. Maldonado F. Ravaglia C. Wells A. U. Colby T. V. Tomassetti S.. (2018). Transbronchial cryobiopsies for the diagnosis of diffuse parenchymal lung diseases: expert statement from the cryobiopsy working group on safety and utility and a call for standardization of the procedure. Respiration 95 (3), 188–200. doi: 10.1159/000484055 29316560

[B8] Hongbin C. Yuyao Y. Hua G. Yifan G. Zhao D. Xiaojuan W.. (2020). Clinical utility of in-house metagenomic next-generation sequencing for the diagnosis of lower respiratory tract infections and analysis of the host immune response. Clin. Infect. Dis. 71 (Supplement_4), S416–SS26. doi: 10.1093/cid/ciaa1516 33367583

[B9] Hutchinson J. P. McKeever T. M. Fogarty A. W. Navaratnam V. Hubbard. R. B. (2016). Surgical lung biopsy for the diagnosis of interstitial lung disease in England: 1997-2008. Eur. Respir. J. 48 (5), 1453–1461. doi: 10.1183/13993003.00378-2016 27660509

[B10] Jiang J. Yang W. Wu Y. Peng W. Zhang W. Pan P.. (2022). Metagenomic next-generation sequencing for identifying pathogens in patients with rheumatic diseases and diffuse pulmonary lesions: a retrospective diagnostic study. Front. Cell Infect. Microbiol. 12. doi: 10.3389/fcimb.2022.963611 PMC9471190 36118036

[B11] Kifjak D. Leitner J. Ambros R. Heidinger B. H. Milos R. I. Beer L.. (2022). Röntgenbefunde bei diffusen parenchymatösen lungenerkrankungen [Chest radiography findings in diffuse parenchymal lung diseases]. Radiologe 62 (2), 130–139. doi: 10.1007/s00117-021-00955-8 34997260 PMC8740870

[B12] Kropski J. A. Pritchett J. M. Mason W. R. Sivarajan L. Gleaves L. A. Johnson J. E.. (2013). Bronchoscopic cryobiopsy for the diagnosis of diffuse parenchymal lung disease. PloS One 8 (11), e78674. doi: 10.1371/journal.pone.0078674 24265706 PMC3827078

[B13] Lentz R. J. Taylor T. M. Kropski J. A. Sandler K. L. Johnson J. E. Blackwell T. S.. (2018). Utility of flexible bronchoscopic cryobiopsy for diagnosis of diffuse parenchymal lung diseases. J. Bronchology Interv Pulmonol 25 (2), 88–96. doi: 10.1097/LBR.0000000000000401 28796717 PMC5803464

[B14] Li G. Huang J. Li Y. Feng J. (2020). The value of combined radial endobronchial ultrasound-guided transbronchial lung biopsy and metagenomic next-generation sequencing for peripheral pulmonary infectious lesions. Can. Respir. J. 2020, 2367505. doi: 10.1155/2020/2367505 32322324 PMC7165338

[B15] Liu N. Kan J. Cao W. Cao J. Jiang E. Zhou Y.. (2019). Metagenomic next-generation sequencing diagnosis of peripheral pulmonary infectious lesions through virtual navigation, radial EBUS, ultrathin bronchoscopy, and ROSE. J. Int. Med. Res. 47 (10), 4878–4885. doi: 10.1177/0300060519866953 31436107 PMC6833387

[B16] Ma W. Zhao Y. Lu X. Zhang L. Ma X. Gao J.. (2022). Negative results of bronchoalveolar lavage fluid metagenomic next-generation sequencing in critically ill patients. Front. Cell Infect. Microbiol. 12. doi: 10.3389/fcimb.2022.962283 PMC9640831 36389134

[B17] Matsumoto M. Awano H. Ogi M. Tomioka K. Unzaki A. Nishiyama M.. (2016). A pediatric patient with interstitial pneumonia due to enterovirus D68. J. Infect. Chemother. 22 (10), 712–715. doi: 10.1016/j.jiac.2016.03.009 27118532

[B18] Medicine Branch of Interventional Respiratory Society Chinese Thoracic Diseases Working Committee of Interventional Respiratory Association Respiratory Physicians Branch of Chinese Medical Doctor (2019). Expert consensus on transbronchial cryobiopsies. Chin. J. Tuberc Respir. Dis. 42 (6), 405–412. doi: 10.3760/cma.j.issn.1001-0939.2019.06.002 31189225

[B19] Meyer. Diagnosis K. C. (2014). And management of interstitial lung disease. Transl. Respir. Med. 2, 4. doi: 10.1186/2213-0802-2-4 25505696 PMC4215823

[B20] Mikolasch T. A. Garthwaite H. S. JC Porter (2017). Update in diagnosis and management of interstitial lung disease. Clin. Med. (Lond) 17 (2), 146–153. doi: 10.7861/clinmedicine.17-2-146 28365626 PMC6297625

[B21] Molin L. J. Steinberg J. B. Lanza L. A. (1994). VATS increases costs in patients undergoing lung biopsy for interstitial lung disease. Ann. Thorac. Surg. 58 (6), 1595–1598. doi: 10.1016/0003-4975(94)91638-1 7979720

[B22] Qian M. Zhu B. Zhan Y. Wang L. Shen Q. Zhang M.. (2022). Analysis of negative results of metagenomics next-generation sequencing in clinical practice. Front. Cell Infect. Microbiol. 12. doi: 10.3389/fcimb.2022.892076 PMC9149223 35651750

[B23] Radu D. M. Macey J. Bouvry D. Seguin A. Valeyre D. Martinod E. (2012). Biopsie pulmonaire chirurgicale: indications et incidences thérapeutiques [Surgical lung biopsy: indications and therapeutic implications]. Rev. Pneumol Clin. 68 (2), 161–169. doi: 10.1016/j.pneumo.2012.01.011 22425502

[B24] Ravaglia C. Wells A. U. Tomassetti S. Gurioli C. Gurioli C. Dubini A.. (2019). Diagnostic yield and risk/benefit analysis of trans-bronchial lung cryobiopsy in diffuse parenchymal lung diseases: a large cohort of 699 patients. BMC Pulm Med. 19 (1), 16. doi: 10.1186/s12890-019-0780-3 30651103 PMC6335717

[B25] Ronaghi R. Oh S. (2022). Transbronchial lung cryobiopsy for diffuse parenchymal lung disease. Semin. Respir. Crit. Care Med. 43 (4), 536–540. doi: 10.1055/s-0042-1748918 35777417

[B26] Rubenfeld G. D. Caldwell E. Peabody E. Weaver J. Martin D. P. Neff M.. (2005). Incidence and outcomes of acute lung injury. N Engl. J. Med. 353 (16), 1685–1693. doi: 10.1056/NEJMoa050333 16236739

[B27] Saraya T. Kimura H. Kurai D. Tamura M. Ogawa Y. Mikura S.. (2018). Clinical significance of respiratory virus detection in patients with acute exacerbation of interstitial lung diseases. Respir. Med. 136, 88–92. doi: 10.1016/j.rmed.2018.02.003 29501253 PMC7125637

[B28] Sato T. Teramukai S. Kondo H. Watanabe A. Ebina M. Kishi K.. (2014). Impact and predictors of acute exacerbation of interstitial lung diseases after pulmonary resection for lung cancer. J. Thorac. Cardiovasc. Surg. 147 (5), 1604–11.e3. doi: 10.1016/j.jtcvs.2013.09.050 24267779

[B29] Song J. W. Hong S. B. Lim C. M. Koh Y. Kim D. S. (2011). Acute exacerbation of idiopathic pulmonary fibrosis: incidence, risk factors and outcome. ur Respir. J. 37 (2), 356–363. doi: 10.1183/09031936.00159709 20595144

[B30] Sun H. Wang F. Zhang M. Xu X. Li M. Gao W.. (2022). Diagnostic value of bronchoalveolar lavage fluid metagenomic next-generation sequencing in pneumocystis jirovecii pneumonia in non-HIV immunosuppressed patients. Front. Cell Infect. Microbiol. 12. doi: 10.3389/fcimb.2022.872813 PMC9024294 35463643

[B31] Travis W. D. Costabel U. Hansell D. M. King T. E. Jr. Lynch D. A. Nicholson A. G.. (2013). An official American thoracic Society/European respiratory society statement: update of the international multidisciplinary classification of the idiopathic interstitial pneumonias. Am. J. Respir. Crit. Care Med. 188 (6), 733–748. doi: 10.1164/rccm.201308-1483ST 24032382 PMC5803655

[B32] Ueno H. Hayashi M. Nagumo S. Ichikawa K. Aoki N. Ohshima Y.. (2021). Disseminated varicella-zoster virus infection causing fatal pneumonia in an immunocompromised patient with chronic interstitial pneumonia. Intern. Med. 60 (7), 1077–1082. doi: 10.2169/internalmedicine.5396-20 33162474 PMC8079903

[B33] Vanfleteren L. E. Linssen C. F. (2010). Role of microorganisms in interstitial lung disease. Curr. Opin. Pulm Med. 16 (5), 489–495. doi: 10.1097/MCP.0b013e32833b1c54 20592601

[B34] Vassallo R. (2003). Viral-induced inflammation in interstitial lung diseases. Semin. Respir. Infect. 18 (1), 55–60. doi: 10.1053/srin.2003.50008 12652455

[B35] Wei Gu Steve M. Chiu Charles Y. (2019). Clinical metagenomic next-generation sequencing for pathogen detection. Annu. Rev. Pathology: Mech. Dis. 14, 319–338. doi: 10.1146/annurev-pathmechdis-012418-012751 PMC6345613 30355154

[B36] Xu. Distribution characteristics A. (2020). And drug resistance of pathogens in patients with infectious interstitial pneumonia. J. Clin. Med. Pract. 24 (19), 26–8,33. doi: 10.7619/jcmp.202019008

[B37] Ye Z. Chen J. Chen M. Wu J. (2020). Is the interstitial lung disease induced by trastuzumab? case report and literature review. J. Clin. Pharm. Ther. 45 (5), 1183–1186. doi: 10.1111/jcpt.13118 31990091

[B38] Yoon S. H. Lee S. M. Park C. H. Lee J. H. Kim H. Chae K. J.. (2020). Clinical practice guideline for percutaneous transthoracic needle biopsy of pulmonary lesions: a consensus statement and recommendations of the Korean society of thoracic radiology. Korean J. Radiol. 22 (2), 263–280. doi: 10.3348/kjr.2020.0137 33236542 PMC7817630

[B39] Zhang Q. Li S. Zhou W. Zheng L. Ren Y. Dong L.. (2021). Application of metagenomic next-generation sequencing (mNGS) combined with rapid on-site cytological evaluation (ROSCE) for the diagnosis of chlamydia psittaci pneumonia. Int. J. Clin. Exp. Pathol. 14 (4), 389–398. 33936360 PMC8085828

[B40] Zhao H. Wei L. Li H. Zhang M. Cao B. Bian J.. (2021). Appropriateness of antibiotic prescriptions in ambulatory care in China: a nationwide descriptive database study. Lancet Infect. Dis. 21 (6), 847–857. doi: 10.1016/S1473-3099(20)30596-X 33515511

[B41] Zhou H. Ouyang C. Han X. Shen L. Ye J. Fang Z.. (2022). Metagenomic sequencing with spiked-in internal control to monitor cellularity and diagnosis of pneumonia. J. Infect. 84 (1), e13–ee7. doi: 10.1016/j.jinf.2021.09.018 34600934

