# Supplementary material for: Combination of transbronchial cryobiopsy based clinic-radiologic-pathologic strategy and metagenomic next-generation sequencing for differential diagnosis of rapidly progressive diffuse parenchymal lung diseases
Source: Front Cell Infect Microbiol. 2023 Jun 20;13:1204024. doi: 10.3389/fcimb.2023.1204024 (PMC10318139; doi:10.3389/fcimb.2023.1204024)
Supplement: Supplementary file 1 [file Table_1.docx]

**Supplementary table 1**. The chest HRCT imaging features of RP-DPLD patients.

| **Descriptions of imaging characteristics** | **Disease category** | **Value** |
| --- | --- | --- |
| Diffuse nodular shadows with variable sizes in both lungs, consolidation lesions, thickened interlobular septa, subpleural lines, nodular-like irregular thickening of pleural, subpleural honeycomb lesions, traction bronchiectasis, peribronchial and hilar lymphadenopathy enlargement. | Sarcoidosis | 2 |
|  | Pulmonary lymphoma | 2 |
|  | Metastatic malignant tumour of the lung | 13 |
| Diffuse ground-glass opacity in both lungs, centrilobular nodules, local grid shadow with limited traction bronchiectasis. | HP | 10 |
|  | Infection-related RP-DPLD | 17 |
| Exudation and consolidation in multiple lobes of bilateral lungs, irregular nodules, thickened interlobular septa, stromal proliferation was distributed along bronchovascular bundles, pleural effusion. | DLI | 7 |
|  | OP | 3 |
|  | CTD-ILD | 15 |
|  | IPAF | 7 |
| Diffuse distribution of cysts, interstitial hyperplasia. | LAM | 1 |

HRCT, high-resolution computed tomography; RP-DPLD, rapidly progressive diffuse parenchymal lung diseases; HP, hypersensitivity pneumonitis; DLI, drug-induced lung injuries; OP, organizing pneumonia; CTD-ILD, connective tissue disease-related interstitial lung disease; IPAF, interstitial pneumonia with autoimmune features; LAM, lymphangioleiomyomatosis.
