# Supplementary material for: Combination of transbronchial cryobiopsy based clinic-radiologic-pathologic strategy and metagenomic next-generation sequencing for differential diagnosis of rapidly progressive diffuse parenchymal lung diseases
Source: Front Cell Infect Microbiol. 2023 Jun 20;13:1204024. doi: 10.3389/fcimb.2023.1204024 (PMC10318139; doi:10.3389/fcimb.2023.1204024)
Supplement: Supplementary file 2 [file Table_2.docx]

**Supplementary table 2**. Therapeutic strategies before and after the diagnosis of RP-DPLD patients.

| **Group** | **Disease category** | **Disease subtypes** | **Case No.** | **Pre-hospitalization medication** | **Treatment strategies after diagnosis** |
| --- | --- | --- | --- | --- | --- |
| Infection-related RP-DPLD | Infection-induced RP-DPLD |  | 29 |  | corresponding antibiotic treatment |
|  | CTD-ILD co-infection | SLE with SS | 2 | glucocorticoid+CTX+ antibiotics | glucocorticoid+CTX+antibiotics |
|  |  | Vasculitis | 4 | antibiotics | glucocorticoid+antibiotics |
|  |  | DM | 3 | antibiotics | glucocorticoid+antibiotics |
|  |  | Rheumatoid arthritis | 4 | glucocorticoid+azathioprine+antibiotics | glucocorticoid+azathioprine+antibiotics |
|  | IPF co-infection |  | 6 | nintedanib+antibiotics | nintedanib+antibiotics |
| Noninfectious RP-DPLD | CTD-ILD | SS | 2 | antibiotics | glucocorticoid |
|  |  | ASS | 7 | antibiotics | glucocorticoid+CTX |
|  |  | AAV | 2 | antibiotics | glucocorticoid |
|  |  | EPD | 2 | antibiotics | glucocorticoid |
|  |  | IgG4-RD | 1 | antibiotics | glucocorticoid |
|  |  | Sarcoidosis | 2 | antibiotics | glucocorticoid |
|  |  | LAM | 1 | antibiotics | rapamycin |
|  | DLI | Amiodarone-induced lung injury | 3 | antibiotics+amiodarone | glucocorticoid |
|  |  | PD-1 | 3 | antibiotics+camrelizumab | glucocorticoid |
|  |  | Paraquat | 1 | antibiotics | nintedanib |
|  | Metastatic malignant tumor of the lung |  | 13 | antibiotics | anti-neoplastic therapy |
|  | IPAF |  | 12 | antibiotics | glucocorticoid |
|  | HP |  | 10 | antibiotics | glucocorticoid |
|  | OP |  | 3 | antibiotics | glucocorticoid |
|  | Pulmonary lymphoma |  | 2 | antibiotics | anti-neoplastic therapy |
|  | Smoking related ILD |  | 1 | antibiotics | stop smoking+ICS+LABA+LAMA |
|  | Radiation pneumonitis |  | 1 | antibiotics | glucocorticoid |
|  | IPPFE |  | 1 | antibiotics | glucocorticoid |

RP-DPLD, rapidly progressive diffuse parenchymal lung diseases; SLE, systemic lupus erythematosus; SS, Sjögren syndrome; DM, dermatomyositis; IPF, idiopathic pulmonary fibrosis; CTD-ILD, connective tissue disease-related interstitial lung disease; HP, hypersensitivity pneumonitis; ASS, antisynthetase syndrome; AAV, anti-neutrophil cytoplasmic antibody associated vasculitis; EPD eosinophilic pulmonary diseases; IgG4-RD, IgG4 related diseases; LAM, lymphangioleiomyomatosis; IPAF, interstitial pneumonia with autoimmune features; DLI, drug-induced lung injuries; OP, organizing pneumonia; ILD, interstitial lung disease; IPPFE, idiopathic pleuroparenchymal fibroelastosis; CTX, cyclophosphamide; ICS, inhaled corticosteroid; LABA, long-acting β2 agonist; LAMA, long-acting anticholinergics.
